# TMEM33 as a Prognostic Biomarker of Cervical Cancer and Its Correlation with Immune Infiltration

**DOI:** 10.1155/2023/5542181

**Published:** 2023-05-27

**Authors:** Hui Zhang, Jun Wang, Ji Yang, Qingwen He, Sanli Guan, Minxia Qiao, Jian Zhao, Xiu Wang

**Affiliations:** Department of Ultrasound, Xi'an People's Hospital (Xi'an Fourth Hospital), Xi'an, China

## Abstract

In women all over the world, cervical cancer (CC) ranks as the fourth most common form of cancer to be diagnosed. It was previously reported that transmembrane protein 33(TMEM33) could report a poor prognosis in several cancers. The current study is aimed at investigating the potential prognostic value of TMEM33 and its relevance to the tumor microenvironment in CC in a comprehensive manner. In this study, CC specimens presented noticeably higher TMEM33 expression level in comparison to nontumor specimens. In pan-cancer assays, it was found that TMEM33 was present at a high level in many different kinds of tumors. We found that patients with CC patients who had a high TMEM33 expression presented worse overall survival (OS) and disease-free survival (DFS) relative to patients who had a low TMEM33 expression. According to the results of a multivariate analysis, a high level of TMEM33 expression can significantly and independently predict the prognosis of CC. The levels of TMEM33 were found to have a negative correlation with resting dendritic cells, resting mast cells, plasma cells, T cells CD8, T cells regulatory, and regulatory T cells. Finally, we confirmed that TMEM33 was overexpressed in CC cells, and its knockdown distinctly suppressed the proliferation and invasion of CC cells. Overall, we provided evidences that TMEM33 could be used as a potential biomarker to assess the prognosis and the level of immune infiltration in CC.

## 1. Introduction

Cervical cancer (CC) is a representative disease and the third leading cause of death related to cancers among females [1]. In 2018, there were about 570,000 newly diagnosed CC cases worldwide, leading to 311,000 death cases among females [2]. It is well known that infection with the human papilloma virus (HPV) is a common cause of the carcinogenesis of CC in populations with high risks [3, 4]. However, approximately 90% of CC developed in low- and middle-income countries lack screening and HPV vaccination [5]. Patients diagnosed with cervical cancer typically undergo one of two treatment modalities, either surgery or a chemotherapy-radiotherapy combination [6, 7]. The cancer, on the other hand, is almost certainly incurable for the patient. There is a high risk that cervical cancer will progress from an early stage to a more advanced stage if it has not been treated [8, 9]. As a result, diagnosis and treatment at an early stage are extremely important. Future researches are suggested to understand the processes leading to CC development and to locate novel biomarkers capable of aiding in the early diagnosis and treatment of the disease.

Growing evidences suggest that both the intrinsic characteristics exhibited by the tumor cells and components in the tumor microenvironment (TME) can determine the cancer malignancy degree [10]. These components include endothelial cells, immune cells, inflammatory mediators, mesenchymal cells, and extracellular matrix molecules [11]. There is an increasing body of evidence suggesting that the characteristics exhibited by tumor-infiltrating immune cells (TIICs) can impact cancer onset and development [12, 13]. TIIC type and density can predict patients' survival and impact the responses of tumors to treatment [14]. Hence, TIICs can promisingly serve as clinical biomarkers targeting cancers and other malignancy types. Tumor-associated neutrophils (TANs) are the most common type of immune cell. They eliminate pathogens and prevent the host from being infected by microorganism. Additionally, TANs have been shown to present a positive relevance to poorer prognosis in gastric cancer and breast cancer. In addition, tumor-associated macrophages (TAMs) are capable of inhibiting antitumor immunity and promoting tumor progression, as well as having a negative correlation with the prognosis of patients with CC [15, 16]. Also, the TME could impact the gene expression in tumor tissues and contribute to the clinical outcome. All these elucidated the relationship of TME with cancer progression, which opened the door to the possibility of improving the treatment of tumors.

Transmembrane protein 33 (TMEM33) has been conserved throughout the course of evolution. Previous studies found that TMEM33 was a downstream effector of PKM2 and that it regulated the activation of SREBP and lipid metabolism [17]. The depletion of PKM2 resulted in increased TMEM33 expression, which, in turn, promoted SCAP degradation through its interactions with the ubiquitin ligase RNF5. On the other hand, there was limited information regarding the possible function of TMEM33 in tumors. The study was the first one to hypothesize that TMEM33 might be a novel prognostic biomarker involved in TME in CC patients.

## 2. Materials and Methods

### 2.1. Raw Data

TMEM33 expression data together with clinical information was gathered from the TCGA public database (http://cancergenome.nih.gov). This data set included 3 normal tissues and 306 cancerous tissue samples. An HTseq tool was used to compute the level 3 HTSeq-fragments per kilobase per million (FPKM) samples, and these results were then converted to transcripts per million (TPM) units. In addition, we acquired publicly accessible transcript data from the Genotype-Tissue Expression (GTEx) database. This data was consistently maintained by the Toil process from UCSC Xena (https://xenabrowser.net/datapages/). In addition, 292 CC patients were included for survival assays.

### 2.2. TMEM33 Expression Pattern in Human Pan-Cancer

The data of normal tissues from the GTEx database were combined with the data from TCGA in order to investigate the TMEM33 dysregulation that occurs between different cancer types and normal tissues. The TCGA database provided access to the RNA sequencing data and the clinical follow-up information of patients who suffered from 33 different cancer types. All expression data were normalized via log2 conversion.

### 2.3. Survival Analysis

Both the survminer and the survival packages (version 0.4.6; http://cran.r-project.org/) by R software were utilized in the survival analysis that was conducted. We screened out 292 tumor samples out of 309 CC cases considering the following conditions: (i) eliminate samples with a predicted lifespan of less than one month; (ii) eliminate normal samples; (iii) eliminate samples of which the clinical information were incomplete. The Kaplan-Meier method served for generating a survival curve. The log-rank test assisted in determining the statistical significance, and a *p* value cutoff of 0.05 served as the significant threshold.

### 2.4. TICs Profile

CIBERSORT is an algorithm that has applications for discovering biological biomarkers and potential therapeutic targets. It has the capability of discriminating between 22 human immune cell morphologies in a manner that is both extremely sensitive and specific. Chen et al. revealed that by using the support regression vector-based machine learning approach, they were able to show that CIBERSORT efficiently resolves cell subtypes that have comparable gene expression patterns through the use of benchmarking analysis [18]. CIBERSORT was used to estimate the TIC abundance profile in each and every tumor sample [19]. For the subsequent analyses, we only considered those patients eligible whose CIBERSORT *p* values were less than 0.05. The total number of immune cell type fractions estimated for each sample was added up to 1 after being summed.

### 2.5. Cell Lines and Cell Cultures

The human normal epithelial cell line HaCaT and the CC cell lines HeLa, SiHa, C-33A, and CaSKi came from the cell bank of the Chinese Academy of Sciences. These cell lines were used to study CC. These cells were grown in a high-glucose DMEM medium with 10% fetal bovine serum(FBS) in an atmosphere that contained 5% carbon dioxide and humidified at 37 degrees Celsius.

### 2.6. Cell Transfection

Small interfering RNAs (siRNAs) against TMEM33 were provided by Shanghai GenePharma, as were control siRNAs (si-NC). The indicated siRNAs were transfected into cells by using the Lipofectamine 2000 reagent(Invitrogen; Thermo Fisher Scientific, Inc.).

### 2.7. Quantitative RT-PCR

In order to conduct the experiments, RNAse-free water was required. cDNA was synthesized using an RT2 first-strand kit that was purchased from Qiagen in China. After adding and mixing 1 microgram (*μ*g) of RNA and 2 microliters (*μ*l) of genomic DNA elimination mix, the mixture then underwent 5 min of incubation at 42 degrees Celsius, after which it was rapidly transferred to ice-cold water for one minute. Following the addition of the reverse transcription mix, which included a 5 buffer and a reverse transcriptase enzyme, the mixture underwent 15 min of incubation at a temperature of 42 degrees. When the incubation period ended, the tube that contained the reaction mixture was heated to 95 degrees Celsius for terminating the reaction. All of the genes were identified by utilizing probes manufactured by Qiagen. The GAPDH gene served as an internal control to help standardize the results. Primers used for RT-RCR are presented as follows: TMEM33-F: ATGGCAGATACGACCCCGAA, TMEM33-R: GAAAGCCACATTGCCGTGTC. GAPDH-F: 5′-CTGGGCTACACTGAGCACC-3′, GAPDH-R: 5′-AAGTGGTCGTTGAGGGCAATG-3′.

### 2.8. Cell Proliferation Assays

To perform the CCK-8 assays, each well of 96-well plates contained cultured CC cells that had been transfected by either a silencing or control sequence of TMEM33. The total number of cells used was *N* = 1 × 10^4^. In order to determine the health of the cells, a 10% CCK-8 working solution (Dojindo, Japan) was formulated, and then 100 *μ*l of it was injected into each well to receive 2 h of incubation at 37 degrees Celsius. For determining the relative cell viability, the absorbance at 450 nm was utilized.

### 2.9. Cell Clone Formation Experiment

In order to get started with the experiment, HeLa and SiHa cells were transfected and then plated in a 6-well plate at a density of 1 × 10^3^ cells per well. Following a transfection process that lasted for 24 hours, the cells were put into a complete medium and given the chance to develop for 14 days. After the allotted time for incubation had passed, the cells were stained with 0.1% crystal violet and then fixed in paraformaldehyde at a concentration of 4%. After that, a microscope was used to count the colonies, each of which had to have at least 50 cells.

### 2.10. 5-Ethynyl-2′-deoxyuridine (EdU) Assay

After a transfection that lasted for 48 hours, HeLa and SiHa cells were seeded at a density of 1 × 10^4^ cells per well in 96-well plates. These cells were then tagged with the BeyoClickTM EdU cell proliferation kit (Beyotime, Shanghai, China). The 4′,6-diamidino-2-phenylindole (DAPI) staining solution was utilized in order to see the nuclei of the cells. Using a fluorescent microscope(Olympus, Tokyo, Japan), it was possible to see cells that had been positively tagged. In order to guarantee the accuracy of the findings, the experiment was repeated three times with no overlap between the runs.

### 2.11. Transwell Assay

A transwell test was carried out in order to measure the capacity of the cells to invade. In this particular experiment, cells were seeded into the top chambers of 24-well plates that had been precoated with Matrigel (Millipore, MA, USA) and then cultured for 48 hours in serum-free media. Following incubation, the invasive cells were fixed with 4% paraformaldehyde and then stained for 20 minutes with a crystal violet solution containing 0.25% crystal violet (Sigma-Aldrich Co., St. Louis, MO, USA). Following this step, stained cells were seen and counted using an inverted microscope manufactured by Nikon in Japan. Five distinct microscopic images were then chosen at random for examination. With the use of this technology, we were able to evaluate the capability of cells to infiltrate through a barrier, which gives important insight into the metastatic potential of the cells.

### 2.12. Western Blot Analysis

As the first stage in the process of obtaining protein lysates, we utilized RIPA buffer. After that, the total protein samples were separated using SDS-PAGE at a 12.5% concentration and then deposited onto PVDF membranes (Thermo Fisher, IL, USA). Before the membranes were probed with primary antibodies against the target proteins at 4°C for a whole night, they were first treated with 5% nonfat milk for the purpose of preventing any nonspecific binding. Secondary antibodies against TMEM33 and GAPDH (Abcam) were added to the membranes after they had been washed three times with PBS. The membranes were then left to incubate in the dark at room temperature for one hour. A chemiluminescence device was utilized in order to carry out the protein concentration measurement study.

### 2.13. Statistical Analysis

Using the R programming language (R version: 3.6.1), each and every piece of data was analyzed. A *t*-test with two independent hypotheses served for data analysis. The Kaplan-Meier method served for the actuarial calculations needed to determine overall survival rates. The Cox proportional hazard regression model determined the independent prognostic factors. In order to be considered statistically significant, the two-tailed *p* value needed to be lower than 0.05.

## 3. Results

### 3.1. TMEM33 Expression Was Overexpressed in CC and Its Pan-Cancer Analysis

**Figure 1:**
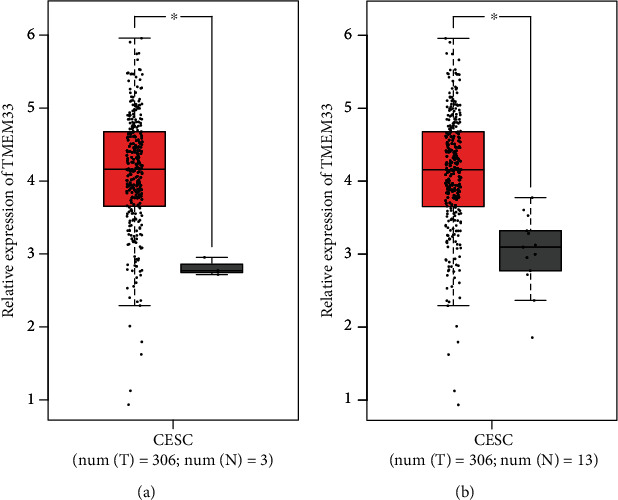
TMEM33 expression in CC tissues and nontumor specimens from (a) TCGA datasets or (b) TCGA datasets and GTEx data.

**Figure 2:**
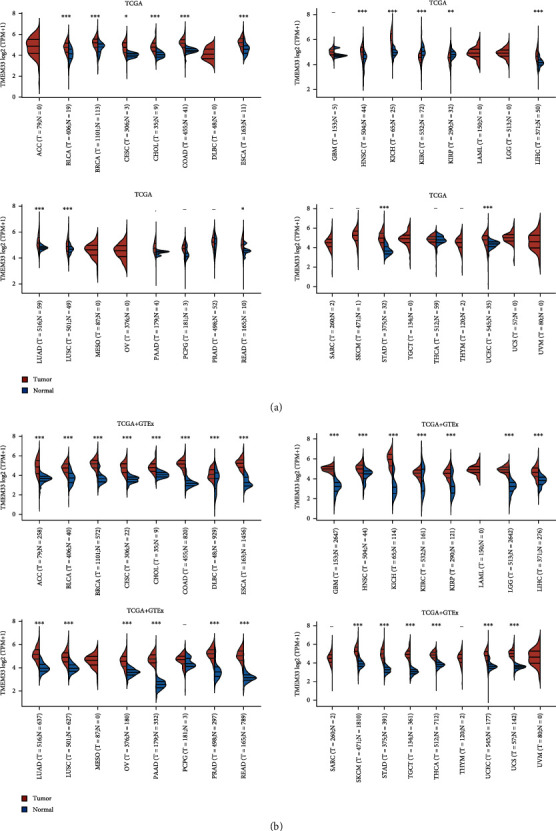
The differential expression of TMEM33 in 33 types of tumors and the nontumor tissues from (a) TCGA datasets or (b) TCGA datasets and GTEx data.

Firstly, we examined the expression of TMEM33 in CC using data from TCGA datasets and GTEx. When compared with nontumor specimens, CC samples presented noticeably higher TMEM33 expression level relative to nontumor samples. This was illustrated in Figures 1(a) and 1(b). After that, we conducted pan-cancer assays and found that TMEM33 was present at a high level in many different kinds of tumors, including ACC, LUAD, and PRAD (Figure 2). Based on our findings, TMEM33 may function as an oncogene in a variety of tumors.

### 3.2. The Prognostic Value of TMEM33 in CC and Pan-Cancer

**Figure 3:**
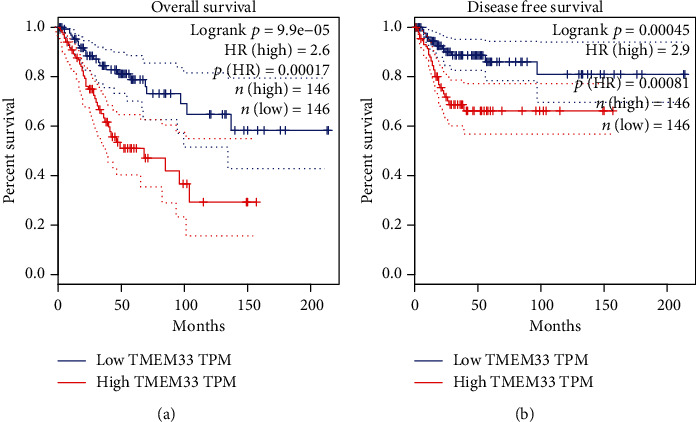
Kaplan-Meier curves of (a) OS and (b) DFS between the TMEM33-high and -low expression cohorts.

**Figure 4:**
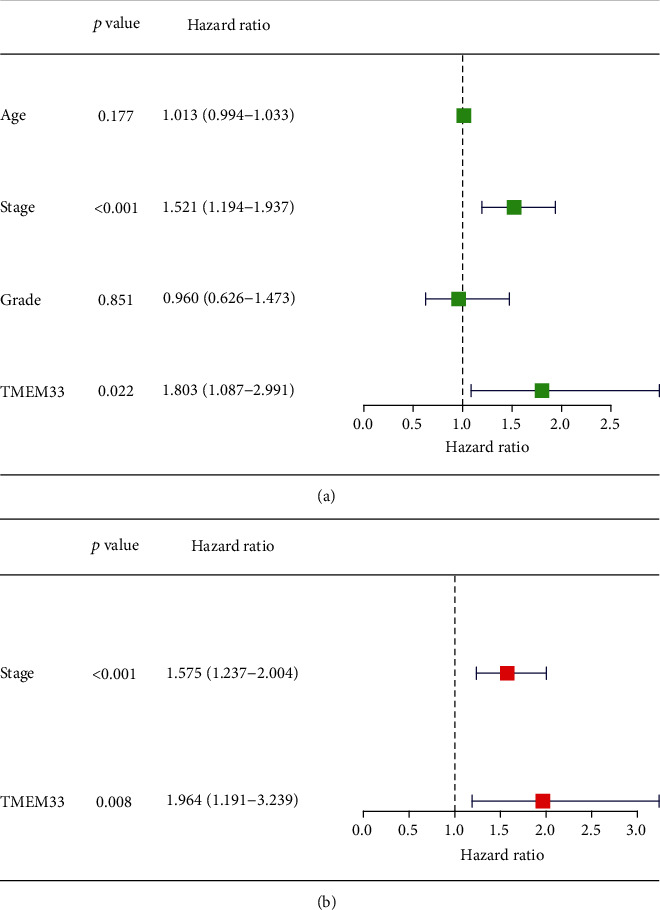
(a) Univariate and (b) multivariate Cox regression analyses of OS in CC patients.

In order to explore the prognostic value of TMEM33 expression in CC, we manually divided CC patients into two groups (high group and low group) based on the mean expression of TMEM33 in CC. The Kaplan-Meier survival analysis was applied to evaluate the prognostic value of TMEM33 expression in patients with CC. We discovered that patients with CC patients with high TMEM33 expression presented a shorter OS (Figure 3(a)) and DFS (Figure 3(b)) relative to CC patients with low TMEM33 expression. A univariate Cox analysis was carried out for the purpose of determining the degree of prognostic significance that clinicopathological factors have for survival rates. Both clinical stage (*p* = 0.001) and TMEM33 expression (*p* = 0.022) had a significant correlation with an individual patient's overall survival (Figure 4(a)). According to the additional multivariate analysis, high TMEM33 expression could significantly and independently predict CC patients' poor OS (hazard ratio [HR] = 1.964, confidence interval [CI] = 1.237 − 2.004) (Figure 4(b)). In addition, the findings of pan-cancer survival assays suggested that TMEM33 expression was linked to the prognosis of patients who were diagnosed with KIRC, SKCM, and CC (Figure S1).

### 3.3. Correlation of TMEM33 with the Proportion of TICs

**Figure 5:**
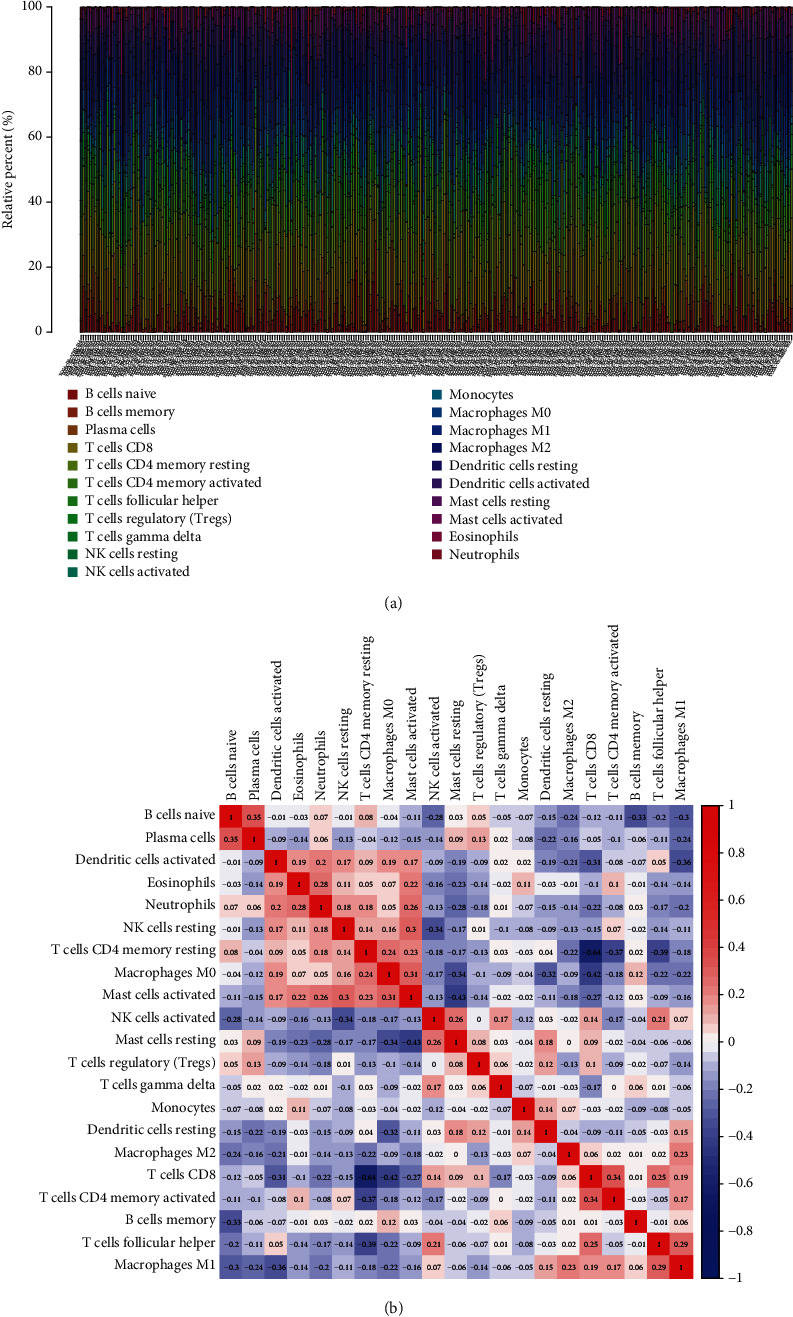
TIC profile in tumor samples together with correlation analysis. (a) Barplot that illustrates the proportion of 21 kinds of TICs in CC tumor samples. (b) Heatmap that illustrates the association of 21 kinds of TICs with numeric in each tiny box.

**Figure 6:**
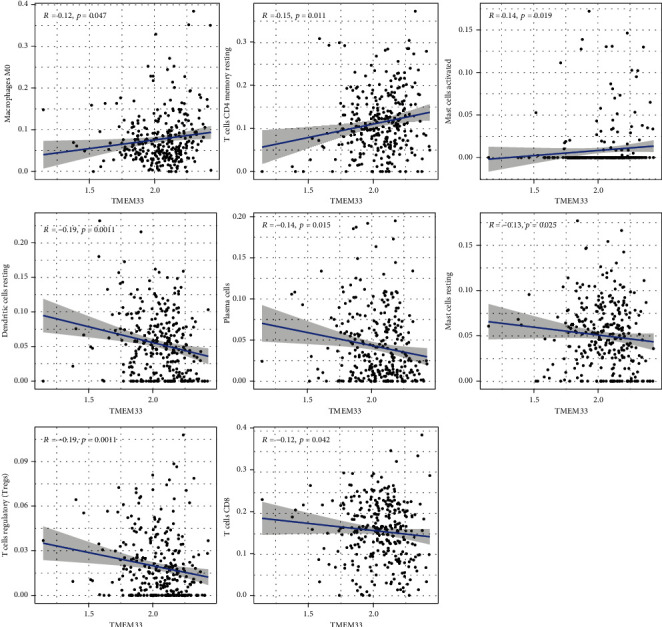
Scatter plot that demonstrates the association between 8 kinds of TICs proportion and the TMEM33 expression.

To more deeply validate that TMEM33 expression was positively correlated with the immune microenvironment, the CIBERSORT algorithm determined the percentage of immune subsets that had infiltrated the tumor. 21 distinct immune cell profiles were generated from CC tissue samples (Figures 5(a) and 5(b)). According to the difference and correlation analyses, 8 kinds of TICs showed a relevance to TMEM33 expression (Figure 6). Thereinto, 3 kinds of TICs presented a positive relevance to TMEM33 expression, including macrophages M0, mast cells activated, and T cells CD4 memory resting; 5 kinds of TICs presented a negative relevance to TMEM33 expression, namely, dendritic cells resting, mast cells resting, plasma cells, T cells CD8, and T cells regulatory (Tregs). All these further proved the impact of TMEM33 levels on TEM immune activity.

### 3.4. The Impact of TMEM33 Knockdown on CC Cell Proliferation and Invasion

**Figure 7:**
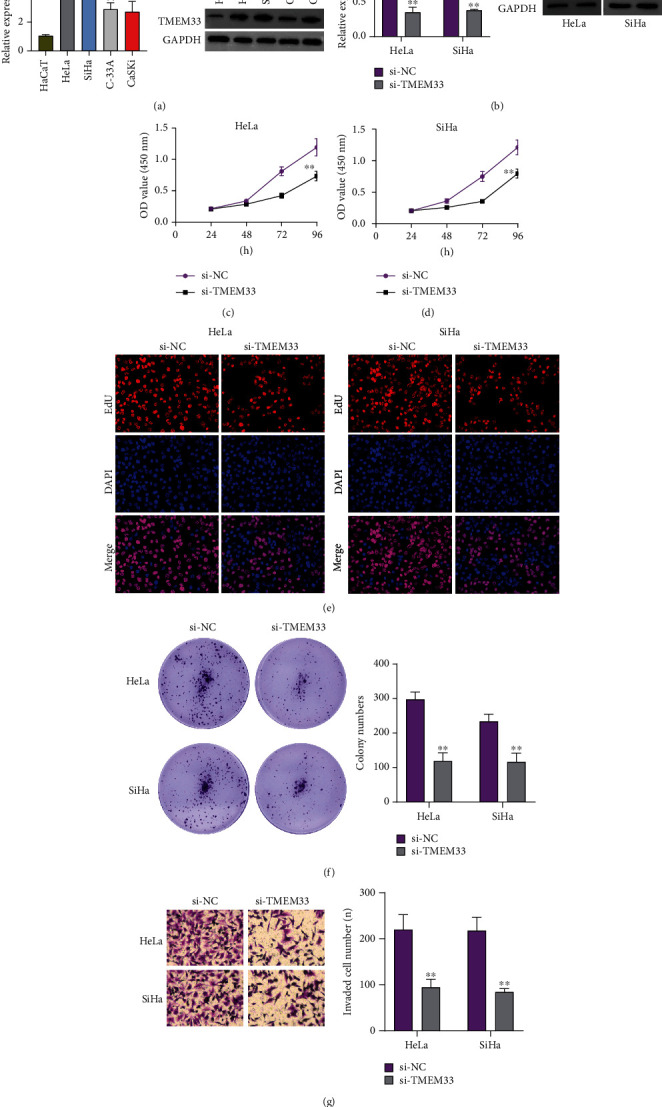
TMEM33 knockdown inhibited CC cell proliferation and invasion. (a) Relative expression of TMEM33 in CC cell lines (HeLa, SiHa, C-33A, and CaSKi) and HaCaT cell line using RT-PCR and western blot. (b) RT-PCR and western blot detected the changes of TMEM33 expressing levels in CC cells after transfection with TMEM33 siRNA. (c, d) CCK8 assays were employed to determine the growth curves of CC cells at 24 h, 48 h, 72 h, and 96 h. (e) EdU immunofluorescence staining assays for HeLa and SiHa cells. (f) Clone formation assays were used to detect cell proliferation in HeLa and SiHa cells. (g) The effect of TMEM33 knockdown1 on the invasion of HeLa and SiHa was determined by transwell assays.

In order to investigate whether or not TMEM33 was expressed in CC, we utilized RT-PCR and western blot on a number of CC cells. In comparison to the HaCaT cells, all four of the CC cells exhibited remarkably higher TMEM33 expression(Figure 7(a)). HeLa and SiHa cells were transfected by small interfering RNAs against TMEM33 (si-TMEM33) for examining the functional roles that TMEM33 plays in CC. Then, RT-PCR and western blot demonstrated that siRNAs had the ability to effectively suppress TMEM33 expression in both HeLa and SiHa cells (Figure 7(b)). According to the CCK-8 assays, TMEM33 siRNA-transfected HeLa and SiHa cells presented remarkably lower optical density (OD 450 nm) relative to cells transfected with si-NC (Figures 7(c) and 7(d)). In addition, the results of EdU staining proved that repressing TMEM33 levels markedly reduced the number of proliferative CC cells (Figure 7(e)). Moreover, clonogenic assays revealed that the clone formation abilities were also attenuated upon TMEM33 knockdown (Figure 7(f)). Finally, we also found that the knockdown of TMEM33 distinctly suppressed the invasion of CC cells (Figure 7(g)).

## 4. Discussion

CC is primarily brought on by an infection with high-risk HPV (hrHPV), and it is the fourth most common cancer type among females around the world [20, 21]. Squamous cell carcinoma (SCC) and adenocarcinoma take up 80-85% and 15-20%, respectively, of all pathological types of cancer that are classified as CC [22, 23]. Even though surgery, chemotherapy, radiation therapy, etc. are available today, recurrence rate and metastasis rate for patients suffering late-stage CC are up to 40.3% and 31%, respectively [24, 25]. Patients who have metastatic CC continue to have a poor prognosis, and the median survival time ranges from 8 to 13 months. As a result, it is of the utmost importance to discover reliable prognostic biomarkers and molecular mechanisms that can impact CC prognosis, which may lead to the discovery of more effective predictive and therapeutic targets.

During our investigation of pan-cancer, we discovered that TMEM33 was overexpressed in two of the tumors. After further investigation, it was found that a higher level of TMEM33 expression reported poorer OS and DFS in patients with CC. In addition, both univariate and multivariate Cox analyses suggested that TMEM33 was a factor that could be considered independent when attempting to forecast the prognosis of patients. All of these results, which have been discussed previously, point to the possibility that TMEM33 is a promising prognostic biomarker for CC patients. In addition, we carried out RT-PCR, which provided further evidence that the level of TMEM33 expression was noticeably elevated in CC cells. According to the results of functional assays, knockdown of TMEM33 suppressed the proliferation and invasion of CC cells. Although a previous study has reported the prognostic value of TMEM33 in CC patients, we firstly provided evidences that TMEM33 may be involved in the progression of metastasis [26]. Our finding suggested TMEM33 as an oncogene in CC progression.

Immunotherapy has only relatively recently been recognized as a potential new treatment option for cancer patients suffering from CC [27, 28]. The tumor microenvironment (TME), which consists of tumor vasculature, stroma cells, the ECM, and various cells of the immune system, has been confirmed to stimulate the developments of various tumors [29]. It is common knowledge that immunosuppressive cells can cause the occurrence of immune escape in TME, that in turn, can promote tumor progression and metastasis [30]. There is evidence that the number of regulatory T cells, or Tregs, a typical immunosuppressive cell type, is correlated with patients' prognosis. This suggests the Treg count as a useful marker for the prognosis of CC. It has been hypothesized that the TME remarkably impacts CC development. TIICs make up the majority of the nontumor components that can effectively assist in assessing CC prognosis. Therefore, it is of the utmost importance to work toward increasing the efficacy of immunotherapy in CC by methodically evaluating the immune properties of the TME and determining the distribution and functions of TIIC. In this study, the TMEM33 expression presented a positive relevance to three different types of TICs, including macrophages M0, activated mast cells, and resting T cells CD4 memory. On the other hand, TMEM33 expression was negatively correlated with five different types of TICs, including resting dendritic cells, resting mast cells, resting plasma cells, resting T cells CD8, and resting T cells regulatory, suggesting TMEM33 may inhibit the infiltration and activation of these immune cells in the tumor microenvironment. This could potentially contribute to tumor immune evasion and promote tumor growth. On the other hand, inhibition of TMEM33 expression could potentially enhance the infiltration and activation of these immune cells, leading to improved antitumor immunity. This suggests that TMEM33 may be a potential therapeutic target for cancer immunotherapy. One potential clinical application related to TMEM33 and immunotherapy is the development of small molecule inhibitors or monoclonal antibodies targeting TEM33. These could be used to enhance the infiltration and activation of immune cells in the tumor microenvironment, potentially improving the efficacy of existing immunotherapies.

The present study has some limitations. First, the clinical information from the TCGA databases was scant and lacked essential details. No in-depth analysis was performed on the information pertaining to neuroimaging, the extent of the resection, radiotherapy, or chemotherapy. Second, additional validation of the prognostic value of TMEM33 expression in patients with CC is required through multicenter, large-scale clinical trials and prospective studies.

## 5. Conclusion

Our research demonstrated that TMEM33 was a candidate biomarker that can predict the outcome of treatment and the patient's prognosis in patients with CC. To elucidate the biological effects and underlying mechanisms of TMEM33, further experimental validation is required.

## Data Availability

Some or all data generated or used during the study can be obtained from the corresponding author upon request.

## Supplementary Materials

Supplementary Materials Figure S1: pan-cancer survival analysis of TMEM33 based on TCGA datasets.
