# Supplementary figures and images for: TMEM33 as a Prognostic Biomarker of Cervical Cancer and Its Correlation with Immune Infiltration
Source: Mediators Inflamm. 2023 May 27;2023:5542181. doi: 10.1155/2023/5542181 (PMC10239303; doi:10.1155/2023/5542181)

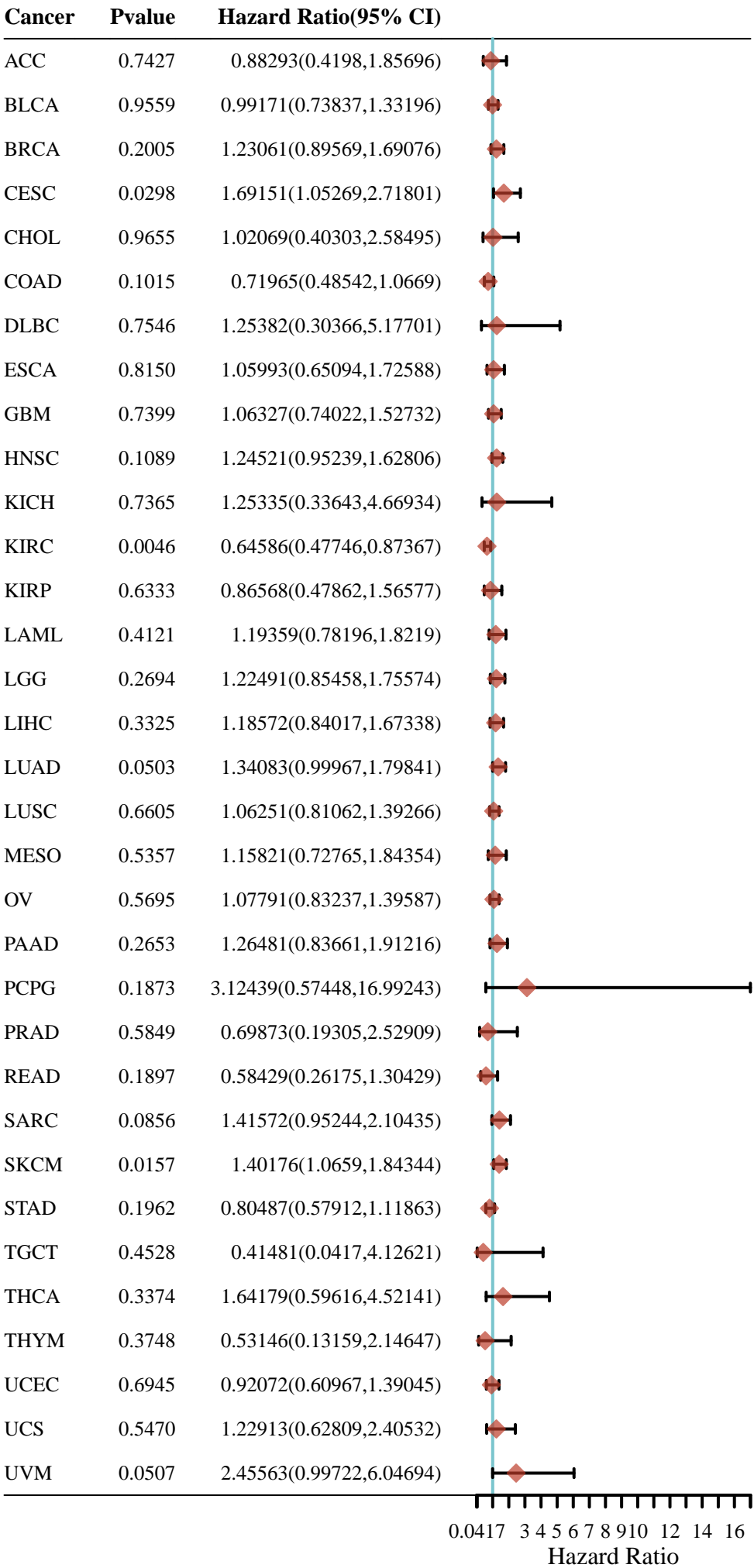

Supplement: Supplementary Materials — Figure S1: pan-cancer survival analysis of TMEM33 based on TCGA datasets. [file 5542181.f11.pdf]
